# Validation of the person-centered maternity care scale in India

**DOI:** 10.1186/s12978-018-0591-7

**Published:** 2018-08-29

**Authors:** Patience A. Afulani, Nadia Diamond-Smith, Beth Phillips, Shreya Singhal, May Sudhinaraset

**Affiliations:** 1 School of Medicine, Institute for Global Health Sciences, University of California, San Francisco, USA; 2 Community Empowerment Lab, Lucknow, India; 3 Jonathan and Karin Fielding School of Public Health, University of California, Los Angeles, USA

**Keywords:** Person-centered care, Maternity care, Measurement, Validation, Developing settings, India

## Abstract

**Background:**

Person-centered care during childbirth is recognized as a critical component of quality of maternity care. But there are few validated tools to measure person-centered maternity care (PCMC). This paper aims to fill this measurement gap. We present the results of the psychometric analysis of the PCMC tool that was previously validated in Kenya using data from India. We aim to assess the validity and reliability of the PCMC scale in India, and to compare the results to those found in the Kenya validation.

**Methods:**

We use data from a cross-sectional survey conducted from August to October 2017 with recently delivered women at 40 government facilities in Uttar Pradesh, India (*N* = 2018). The PCMC measure used is a previously validated scale with subscales for dignity and respect, communication and autonomy, and supportive care. We performed psychometric analyses, including iterative exploratory and confirmatory factor analysis, to assess construct and criterion validity and reliability.

**Results:**

The results provide support for a 27-item PCMC scale in India with a possible score range from 0 to 81, compared to the 30-item PCMC scale in Kenya with a 0 to 90 possible score range. The overall PCMC scale has good reliability (Cronbach alpha = 0.85). Similar to Kenya, we are able to group the items in to three conceptual domains representing subscales for “Dignity and Respect,” “Communication and Autonomy,” and “Supportive Care.” The sub-scales also have relatively good reliability (Cronbach alphas range from 0.67 to 0.73). In addition, increasing scores on the scale is associated with future intentions to deliver in the same facility, suggesting good criterion validity.

**Conclusions:**

This research extends the PCMC literature by presenting results of validating the PCMC scale in a new context. The psychometric analysis using data from Uttar Pradesh, India corroborates the Kenya analysis showing the scale had good content, construct, and criterion validity, as well as high reliability. The overlap in items suggests that this scale can be used across different contexts to compare women’s experiences of care, and to inform and evaluate quality improvement efforts to promote comprehensive PCMC.

## Plain English summary

Disrespect, abuse, and mistreatment during childbirth are commonly reported in both the global scientific community and popular media. Yet till recently, there has been no standardized way to measure women’s experiences of respectful and responsive care, which we refer to as person-centered maternity care (PCMC). In our previous work in Kenya we developed and tested a scale for measuring PCMC, and we wanted to learn how that scale worked in a different setting. We therefore administered the PCMC scale to over 2000 women in India who had recently delivered a baby at a government health facility. To determine if the scale was credible in India, we ran statistical tests to see how the questions in the scale hung or grouped together. The results showed that the PCMC scale worked best in the India sample as 27 questions that gave a holistic measure of women’s experiences during childbirth. Similar to what we found in Kenya, the PCMC scale covered dignity and respect, communication and autonomy, and supportive care. Women with higher PCMC scores were more likely to plan to deliver at the same facility again—implying high credibility. These findings suggest the PCMC scale can be used across many different contexts to compare women’s experiences of maternity care. A standard measurement tool combined with clear policy guidelines can help to improve accountability of facilities, support staff in understanding how to provide person-centered care, and ensure women’s voices, preferences, and values are front and center in the care they receive.

## Background

Person-centered care during childbirth is recognized as a valued dimension of quality of maternal and newborn care [1]. Poor person-centered care during childbirth has, however, increasingly been documented around the globe [2–5]. This recognition has prompted recent recommendations by the World Health Organization (WHO) on intrapartum care for a positive childbirth experience [6]. Person-centered maternity care (PCMC) refers to “maternity care that is respectful of and responsive to individual women and their families’ preferences, needs, and values” [7, 8]. The WHO recommendations highlight respectful maternity care, effective communication, and companionship during labor and childbirth as key dimensions of PCMC that should be provided to every women throughout labor and birth [6]. These recommendations are based on a human rights-based approach, as well as on evidence of the potential impacts of these interventions to reducing maternal morbidity and mortality [6].

Although we conceptualize PCMC as a broader construct which includes respectful maternity care, they highly overlap given the broad domains of respectful maternity care that have been proposed [9]. A recent review that synthesized data from sixty-seven studies from 32 countries identified twelve domains of respectful maternity care: being free from harm and mistreatment; maintaining privacy and confidentiality; preserving women’s dignity; prospective provision of information and seeking of informed consent; ensuring continuous access to family and community support; enhancing quality of physical environment and resources; providing equitable maternity care; engaging with effective communication; respecting women’s choices that strengthen their capabilities to give birth; availability of competent and motivated human resources; provision of efficient and effective care; and continuity of care [10]. These domains greatly overlap with previously identified domains of PCMC, which include dignity, autonomy, privacy/confidentiality, communication, social support, trust, supportive care, and the health facility environment [8, 11].

PCMC is important for increasing demand for facility deliveries, as well as for improving maternal and neonatal health outcomes for facility-based deliveries [12–14]. Facility-based deliveries have increased dramatically in India in the last decade. In 2015–16, about 79% of women in India reported that they delivered in a health facility compared to 39% in 2005–6, representing a two-fold increase [15]. Rates are slightly lower in Uttar Pradesh (the setting for our study), where about 68% of women in 2015–16 reported their last delivery was in a facility (up from 21% in 2005–6) [16]. This dramatic increase in facility deliveries is partly fueled by a cash incentive program, called Janani Suraksha Yojana (JSY), offered to women for delivering in a public facility. However, other factors, such as the relationship with community health workers have also played an important role in decision-making around place of birth [17, 18]. Despite the large increase in facility deliveries, India has not seen the expected reductions in maternal and neonatal mortality. Research exploring the impact of JSY on maternal and neonatal mortality generally has pointed to JSY having no impact on these adverse outcomes [19, 20]. It has been hypothesized that poor quality of care, due in part to overburdened health personnel because of the large increase in the number of deliveries, is contributing to the lack of change in maternal and neonatal health outcomes [20]. Past research in Uttar Pradesh specifically have found poor quality of care for delivery and newborn services, and a high prevalence of unqualified providers [21].

In India, alongside evidence of poor maternal and newborn clinical care is increasing evidence of poor PCMC [22–24]. Research in Uttar Pradesh, the most populous state in India, has documented that between 20 to 57% of women who recently delivered in health facilities reported some form of mistreatment during childbirth [23, 24]. In one study, the most commonly reported forms of poor person-centered care included verbal abuse, requests for bribes, not being allowed a companion, and discrimination [23]. Observational data of deliveries at facilities in Uttar Pradesh confirmed women’s reports of mistreatment [25]. Other studies have documented factors associated with mistreatment, including women’s empowerment, presence of support person, and type of providers [24, 26, 27]. Women were more likely to experience mistreatment if they were less empowered [27], had no companions [26], and received care from nurses compared to physicians or midwives [24]. Research in Uttar Pradesh also found that women who reported mistreatment at the time of delivery were more likely to have a complicated delivery or postpartum complications [24]. All of these studies have used different types of tools focused on measuring mistreatment or disrespect and abuse.

Increased awareness of the problem of poor PCMC has highlighted the need for validated measures for it [2, 28, 29]. Afulani et al. (2017) recently validated the first scale to measure person-centered maternity care in Kenya. This validated scale includes 30 items (indicators or questions) with three sub-scales measuring dignity and respect, communication and autonomy, and supportive care. The scale was validated in two populations in Kenya, one rural and one urban. Validation in one country is important for moving this measurement agenda forward; however, the question remains as to how the measurement of a concept as complex and potentially culturally nuanced as person centered care can be translated from one country to another. To our knowledge, there has been no tool validated to holistically measure person-centered maternity care in India. Thus, as part of facility-based quality improvement project being implemented in Kenya and India, we sought to develop a person-centered maternity care scale that was potentially applicable to multiple settings including Kenya and India. The goal of this paper is to present the results of the psychometric analysis of the same tool validated by Afulani et al. 2017 in Kenya in an Indian population.

## Methods

The development of this scale followed standard procedures for scale development and included the following: (1) Literature review to define the construct of person-centered maternity care and identify domains; (2) Item generation based on existing tools with additional questions; (3) Expert reviews in with experts in Kenya, India (Uttar Pradesh), and the US to assess content validity—whether the items represent all possible indicators relevant to the construct [30]; (4) Cognitive interviews with potential respondents in Kenya and in two public health facilities Uttar Pradesh, India to assess how participants internalize the questions and if the questions were being interpreted as intended. It was also used to evaluate problems with the wording of questions and whether questions are context appropriate and salient [31–33]; (5) Pretesting to finalize the scale items and full survey tool; and (6) Structured interviews in surveys with recently delivered women. Iterative revision of items followed each step. Similarly, the items were first translated into Hindi for the cognitive interviews with iterative translations following each revision. Details of the activities carried out in each of these steps up to finalizing the items for the survey are described in Afulani et al. 2017, where the process towards developing the final items and psychometric analysis based on survey data from Kenya are presented [8].

All study activities in India took place in Uttar Pradesh, a state in northern India. Uttar Pradesh is the most populous state in India (current population of 204.2 million), with 75 districts spread across four culturally and geographically distinct zones. The vast majority of Uttar Pradesh residents are considered rural (77%, Census 2011), though nearly all Uttar Pradesh residents live within 50 km of urban or peri-urban areas. A near final version of the PCMC tool was translated and administered to 867 women at nine government facilities in two districts of Uttar Pradesh, as part of the baseline surveys for a quality improvement intervention. However, minor edits were made to the items after this initial survey to obtain the final set of 38 items that were administered in Kenya and used for the analysis that yielded the 30 item PCMC scale. The 30 items from the Kenya validation, in addition to two other items—whether the woman was asked for bribes and whether she was asked to buy items from outside the facility—which we believed were important to the Indian context from our preliminary work in India, were then translated to Hindi and back translated to ensure accuracy. This set of 32 items was added to the study questionnaire for a cross-sectional study on quality of maternity care in Uttar Pradesh, and pre-tested with 10 recently delivered women at Lucknow District Women’s Hospital in June 2017.

The final questionnaire was then administered to 2018 women in 40 high volume public health facilities in 20 districts of Uttar Pradesh. This data is used for the psychometric analysis presented in this paper. The survey was conducted from August through October 2017. Respondents were women aged 18 to 46 years who delivered in the 48 h preceding the survey at any of the 40 participating health facilities. Eligible women were identified by facility staff and subsequently invited by study staff to participate in the survey. Recruitment and consenting took place at the post-natal ward, and respondents were given the option of continuing with the interview in a private space at the facility or at their bed. Most interviews (2015 out of 2018) occurred on the post-natal ward at the patient’s bed. All interviews were conducted in Hindi. All participants provided written informed consent after receiving information about the research. Interviews were conducted using the CommCare platform on tablets, with data uploaded to the server at the end of each day. About fifty women were interviewed per facility depending on facility delivery load (per the study design). Ethical approval for this study was provided by the ethics review boards of University of California, San Francisco and the Community Empowerment Lab in India.

### Psychometric analyses

The psychometric analysis followed the same process as in the Kenya PCMC validation [8].We first examined the distributions of all the items. In instances where questions had responses in the “not applicable” category, we converted the “not applicable” category into the highest category to obtain a uniform scale for the psychometric analysis. This approach is conservative as it assumes the highest quality rating for each “not applicable” response. Only one item (explaining medications) had to be recoded in this format. We also reverse coded negative items in order for responses to reflect a scale of 0 as the lowest level to 3 as the highest level.

We used iterative exploratory and confirmatory factor analysis to assess construct validity—the degree to which the items represent the underlying conceptual structure. Kaiser-Meyer-Olkin (KMO) value of 0.5 or above was used as the criterion for sampling adequacy [34]. The Eigenvalues (the amount of information captured by a factor) and scree plots (plots of Eigenvalues) were used to determine the number of factors to extract. We used both Kaiser’s rule of retaining only factors with eigenvalues exceeding unity and the “break” in the scree plot to decide on how many factors to retain [30, 35, 36]. We then conducted subsequent factor analysis and examined the item loadings to determine which items to retain or delete. Since most of the items had been vetted in the Kenya validation and the goal was not item reduction at this stage, we used a relaxed cut-off of 0.1 to retain items in this analysis [37]. We used oblique rotation, which, allows for correlation between the rotated factors and aligns the factor axes as closely as possible to the groups of the original variables [31, 34, 35]. We compared the factor structure to that obtained in the Kenya validation and tested our final factor structure with confirmatory factor analysis.

We assessed criterion validity—whether the measure is related to other measures or outcomes in theoretically predictable ways—by regressing the final scale on whether not the woman responded she would deliver in the same facility if she were to have another baby [30, 38]. We assessed the internal consistency reliability using Cronbach’s alpha, with Cronbach’s alpha of 0.7 or higher generally considered sufficient evidence of reliability [35]. We used STATA version 14 to perform the statistical analyses.

## Results

We performed the psychometric analysis using data from the full sample (*N* = 2018). Table 1 shows the demographic characteristics of respondents. The average age of the women was about 25 years (range of 18 to 46) with an average parity of 2 (range of 1 to 8 children). Almost all (99%) of the women were married, and 34% had less than primary education. Nearly 85% of the sample resided in the rural portions of the districts.

**Table 1:** Distribution of selected demographic variables (*N* = 2018)

Variables	No.	%	
Age: Mean (SD)	2018	25	(4)
Parity: Mean (SD)	2018	2.2	(1.3)
Marital status			
Married	2013	99.8	
Widowed	2	0.1	
Divorced/Separated	3	0.2	
Education			
No school/Primary	941	46.6	
Post-primary/vocational/Secondary	828	41	
College or above	249	12.3	
Employed			
No	1905	94.4	
Yes	113	5.6	
Pregnancy complications			
No	425	21.1	
Yes	1593	78.9	
Religion			
Christian	1	0.1	
Muslim/other	342	17	
Hindu	1675	83	
Caste			
Scheduled caste/scheduled tribe	574	28.4	
General	332	16.5	
Other backwards caste	1112	55.1	
Total	2018	100	

**Fig. 1:**
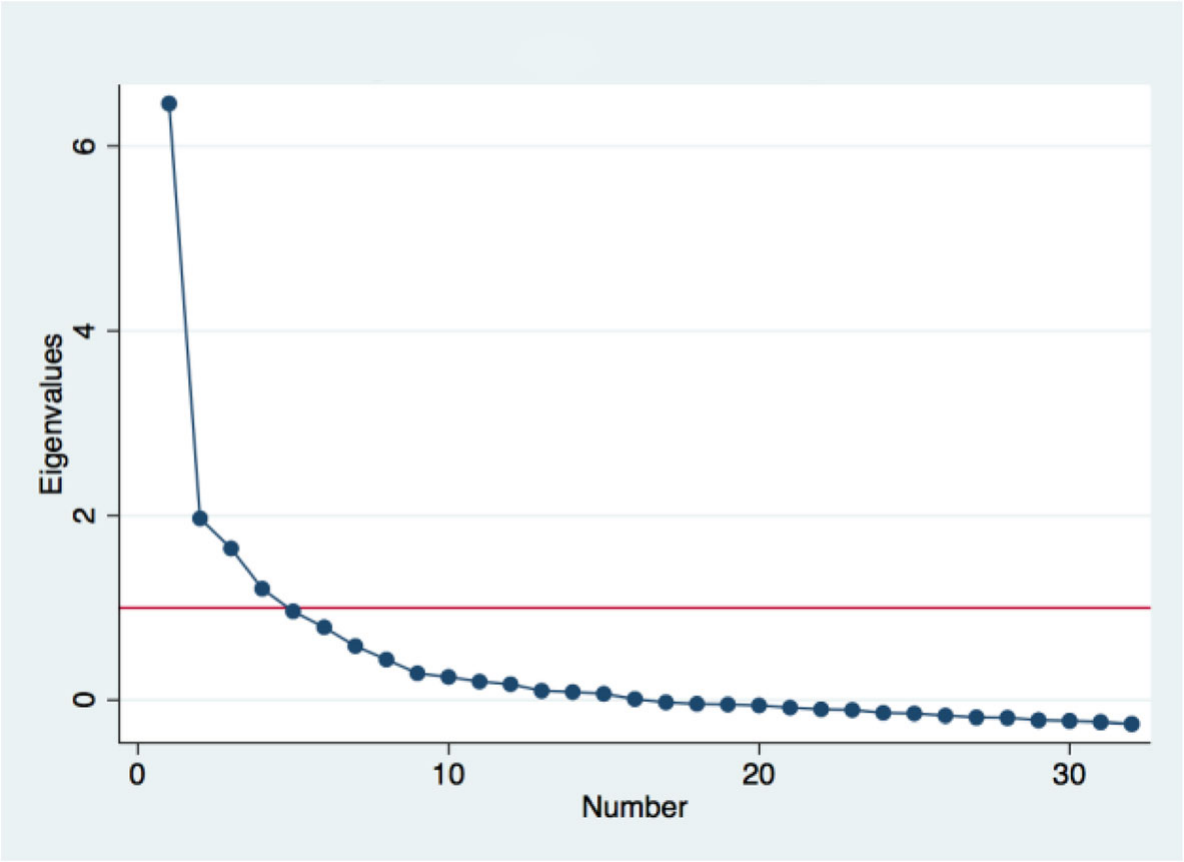
Scree plot for 32 items after exploratory factor analysis

Table 2 shows the original domains, the questions for each domain, and comments on decisions taken related to that item. The distributions for the items are shown in Appendix. The Kaiser-Meyer-Olkin (KMO) measure of sampling adequacy for all items are greater than 0.5, with an overall KMO of 0.91, indicating that overall the variables are satisfactory for factor analysis. The initial exploratory factor analysis with all 32 items yielded four factors (Fig. 1) accounting for 87% of the total variance, although the scree plot showed only one dominant factor. Also, the third and fourth factors in the un-rotated solution did not have any items loading positively on them suggesting a two-factor solution. When the oblique rotation was applied, only the items on labor and delivery support loaded on the fourth factor. When reduced to three factors (i.e. applying the Kenya three-factor structure), only labor and delivery support still loaded on the third factor. For the two-factor solution, 16 items loaded on the first factor and 11 on the second factor. These 27 items also loaded on the single factor. Four items that had factor loadings of less than 0.1 on the retained factors at each stage were dropped. These items are: “Was there clean drinking water available in the facility?”, “Thinking about the labor and postnatal wards, did you feel the health facility was crowded?” and “Were you or your family asked to buy anything from outside the health facility for your care?”. We also dropped the item on “Did the doctors, nurses or other staff at the facility support your anxieties and fears” because the question was identified as ambiguous based on feedback on the original scale.

**Table 2:** Items for person-centered maternity care scale

Original Domain	Question	Referred to in text as	Comment
Dignity/Respect	1. How did you feel about the amount of time you waited? Would you say it was very short, just a little long, somewhat long, or very long?	Time to care	Retained but loads at less than 0.3
Dignity/Respect	2. During your time in the health facility did the doctors, nurses, or other health care providers introduce themselves to you when they first came to see you?	Introduce self	Retained but loads at less than 0.3
Dignity/Respect	3. Did the doctors, nurses, or other health care providers call you by your name?	Called by name	Retained
Dignity/Respect	4. Did the doctors, nurses, or other staff at the facility treat you with respect?	Treated with respect	Retained
Dignity/Respect	5. Did the doctors, nurses, and other staff at the facility treat you in a friendly manner?	Friendly	Retained
Dignity/Respect	6. Did you feel the doctors, nurses, or other health providers shouted at you, scolded, insulted, threatened, or talked to you rudely?	Verbal abuse	Retained
Dignity/Respect	7. Did you feel like you were treated roughly like pushed, beaten, slapped, pinched, physically restrained, or gagged?	Physical abuse	Retained but loads at less than 0.3
Privacy/Confidentiality	8. During examinations in the labor room, were you covered up with a cloth or blanket or screened with a curtain so that you did not feel exposed?	Visual privacy	Retained
Privacy/Confidentiality	9. Do you feel like your health information was or will be kept confidential at this facility?	Record confidentiality	Retained
Autonomy	10. Did you feel like the doctors, nurses or other staff at the facility involved you in decisions about your care?	Involvement in care	Retained
Autonomy	11. Did the doctors, nurses or other staff at the facility ask your permission/consent before doing procedures and examinations on you?	Consent to procedures/exams	Retained
Autonomy	12. During the delivery, do you feel like you were able to be in the position of your choice?	Delivery position choice	Retained
Communication	13. Did the doctors, nurses or other staff at the facility speak to you in a language you could understand?	Language	Retained but loads at less than 0.3
Communication	14. Did the doctors and nurses explain to you why they were doing examinations or procedures on you?	Explain exams/procedures	Retained
Communication	15. Did the doctors and nurses explain to you why they were giving you any medicine?	Explain medicines	Retained
Communication	16. Did you feel you could ask the doctors, nurses or other staff at the facility any questions you had?	Able to ask questions	Retained but loads at less than 0.3
Social Support	17. Were you allowed to have someone you wanted (outside of staff at the facility, such as family or friends) to stay with you during labor?	Labor support	Retained
Social Support	18. Were you allowed to have someone you wanted to stay with you during delivery?	Delivery support	Retained
Supportive Care	19. Did the doctors and nurses at the facility talk to you about how you were feeling?	Talk about feeling	Retained
Supportive Care	20. Did the doctors, nurses or other staff at the facility support your anxieties and fears?	Support anxiety	Deleted based on wording of question
Supportive Care	21. Do you feel the doctors or nurses did everything they could to help control your pain?	Control pain	Retained
Supportive Care	22. When you needed help, did you feel the doctors, nurses or other staff at the facility paid attention?	Attention when need help	Retained
Trust	23. Did you feel the doctors, nurses or other staff at the facility took the best care of you?	Took best care	Retained
Trust	24. Did you feel you could completely trust the doctors, nurses or other staff at the facility with regards to your care?	Trust	Retained
Facility environment	25. Do you think there was enough health staff in the facility to care for you?	Enough staff	Retained
Facility environment	26. Thinking about the labor and postnatal wards, did you feel the health facility was crowded?	Crowded	Deleted: low loading
Facility environment	27. Thinking about the wards, washrooms and the general environment of the health facility, will you say the facility was very clean, clean, dirty, or very dirty?	Clean	Retained but loads at less than 0.3
Facility environment	28. Was there clean drinking water available in the facility?^a^	Water	Deleted: low loading
Facility environment	29. Was there electricity in the facility?	Electricity	Deleted: low loading
Facility environment	30. In general, did you feel safe in the health facility?	Safe	Retained but loads at less than 0.3
Predictability & transparency of payments	31. Did the doctors, nurses or other staff at the facility ask you or your family for money other than the official cost?^b^	Bribe	Retained but loads at less than 0.3
Predictability & transparency of payments	32. Were you or your family asked to buy anything from outside the health facility for your care?^b^	buy supplies	Deleted: low loading

Notes: All items retained loaded at > 0.1 on the final main scale

^a^Question asked as was there water in Kenya version (drinking water added by India survey team)

^b^All items in scale validated from Kenya data except these two questions

**Fig. 2:**
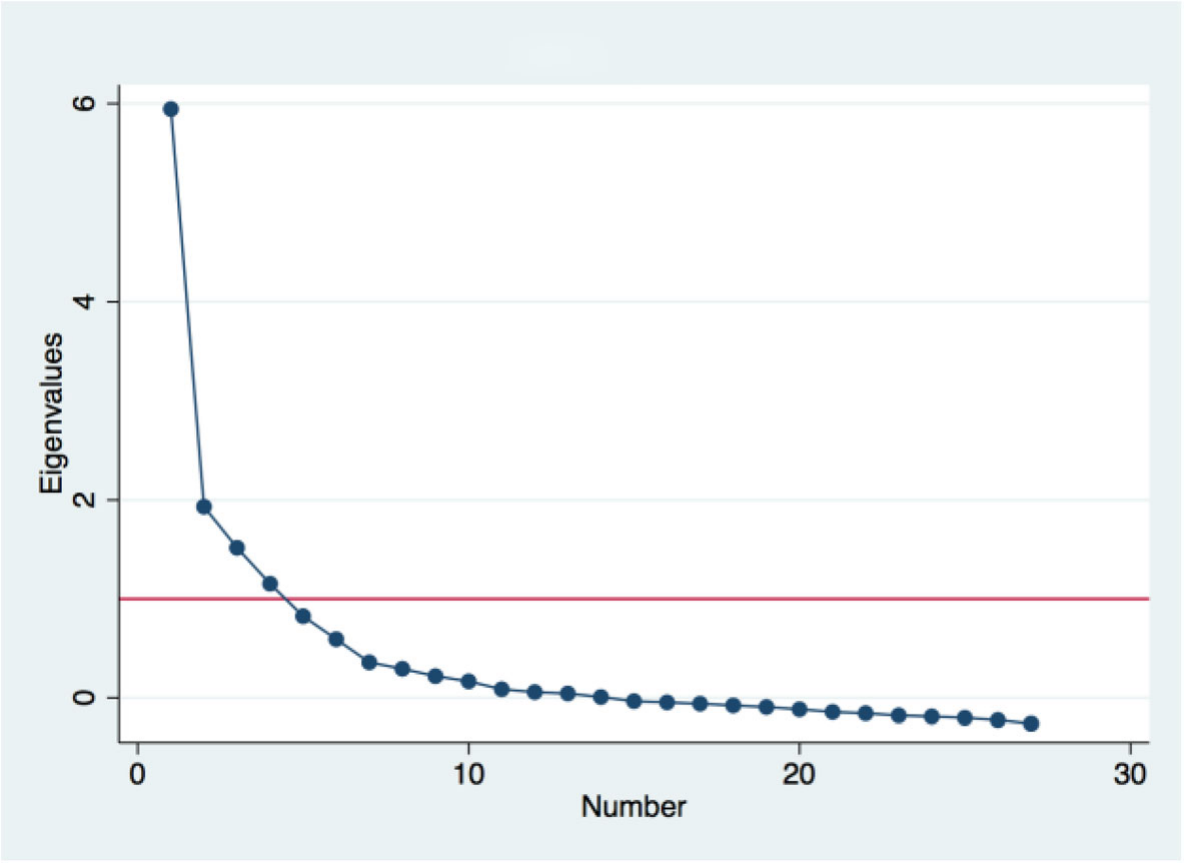
Scree plot for 27 items after exploratory factor analysis

Factor analysis of the remaining 27 items still yielded four factors with one dominant factor (Fig. 2), with item distributions on the factors as in the full set. However, all the items had loadings of > 0.1 (with most greater than 0.3) on the retained factors, including when the factors were constrained to a single factor (Table 3). This suggests high construct validity for a single dominant 27 item PCMC scale based on the India data. However, because the domains of PCMC are overlapping, the items loading on the two factors (which was the best multiple factor solution for the India data) did not represent clear conceptual domains. For instance, the first factor includes items from the domains dignity and respect and supportive care. It however also includes the items on “being spoken to in a language they understand” and “being able to ask questions,” which conceptually should have loaded on the second factor, which has more items on communication and autonomy (but also includes the items on privacy and confidentiality). We therefore regrouped the retained items into three conceptual domains as in the Kenya analysis, to provide the sub-scales for Dignity and respect, Communication and autonomy, and Supportive care. These are theoretically derived categories rather than data driven. However, when factor analysis is run on each set, the items load well on the factor representing each domain, except for the question on “being able to ask questions” which loads negatively on the communication and autonomy domain and positively on the dignity and respect domain, although conceptually it should group under communication and autonomy (Table 4).

**Table 3:** Rotated factor loadings of items on dominant factor for main scale (*N* = 2018)

Variable	Factor loadings
Time to care	0.26
Introduce self	0.12
Called by name	0.42
Treated with respect	0.77
Friendly	0.79
Visual privacy	0.48
Record confidentiality	0.34
Involvement in care	0.63
Consent to procedures/exams	0.42
Delivery position choice	0.38
Language	0.17
Explain exams/procedures	0.48
Explain medicines	0.57
Talk about feeling	0.51
Able to ask questions	0.21
Labor support	0.37
Delivery support	0.35
Attention when need help	0.63
Control pain	0.68
Verbal abuse	0.47
Physical abuse	0.20
Enough staff	0.55
Took best care	0.71
Trust	0.45
Bribe	0.16
Clean	0.12
Safe	0.20

**Table 4:** Rotated factor loadings of items on dominant factor for sub-scales (*N* = 2018)

Subscale	Variable	Factor loadings
Dignity and respect		
	Treated with respect	0.92
	Friendly	0.92
	Verbal abuse	0.51
	Physical abuse	0.25
	Visual privacy	0.43
	Record confidentiality	0.30
Communication and autonomy		
	Introduce self	0.18
	Called by name	0.25
	Involvement in care	0.68
	Consent to procedures	0.70
	Delivery position choice	0.35
	Language	0.17
	Explain exams/ procedures	0.80
	Explain medicines	0.75
	Able to ask questions	−0.05
Supportive care		
	Time to care	0.25
	Labor support	0.54
	Delivery support	0.52
	Talk about feeling	0.42
	Attention when need help	0.67
	Took best care	0.76
	Control pain	0.68
	Trust	0.49
	Safe	0.26
	Enough staff	0.64
	Clean	0.05
	bribe	0.19

The overall PCMC scale has a Cronbach’s alpha of 0.85, suggesting good reliability. The sub-scales also have relatively good reliability (alphas from 0.67 to 0.73 in Table 5). The average PCMC score for the sample based on the sum of the 27 items is 50 (where 0 is the minimum possible score and 81 is the maximum possible score (range for the sample is 16 to 79). In addition, increasing scores on the scale is associated with future intentions to deliver in the same facility (Table 6), suggesting good criterion validity.

**Table 5:** Reliability and distribution of Full PCMC scale and sub-scales (*N* = 2018)

	Alpha	Mean	SD	Min	Max	Possible range
Full PCMC Scale (27 items)	0.85	50.3	10.9	16	79	0–81
Sub-scales						
Dignity and respect (6 items)	0.70	14.1	3.48	2	18	0–18
Communication and autonomy (9 items)	0.67	9.6	4.3	0	25	0–27
Supportive Care (12 items)	0.73	26.7	5.19	6	36	0–36

**Table 6:** Bivariate linear regression of person-centered maternity care score on future intentions to deliver in the same facility (*N* = 2018)

	Coef.	*p*-value	[95% Conf.	Interval]
Will deliver in same facility again in future				
No (ref)				
Yes	16.99	< 0.00	14.30	19.68
Cons	33.87	< 0.00	31.22	36.51

## Discussion

Person-centered care is a recognized dimension of quality of care; however, until recently, there has not been a validated scale for person-centered care for maternity services in low resource settings [8]. This paper extends the PCMC literature by presenting the results of validating the PCMC scale in a new context –India. The analysis provides support for a 27-item multidimensional PCMC scale in Uttar Pradesh, India, in comparison to the 30-item scale derived from the Kenya validation of the same scale. The possible range of scores for the 27-item scale is therefore from 0 to 81 (compared to 0 to 90 for the 30-item Kenya scale). Similar to Kenya, the items can be grouped into three conceptual domains representing subscales for “Dignity and Respect,” “Community and Autonomy,” and “Supportive Care.” These subscales are in general supported by the empirical analysis. The scale has good reliability (Cronbach alpha = 0.85). The subscales also have good reliability (Cronbach alpha ranging from 0.67–0.73), suggesting that future studies may use these individual subscales or the full scale when examining women’s experiences in labor and delivery. The preliminary work towards the development of this scale ensured it had good content validity. The psychometric analysis using data from Uttar Pradesh, India corroborates the Kenya analysis showing the scale had good construct and criterion validity, as well as high reliability.

Validating the PCMC scale in a different context is necessary to highlight potential cultural and social differences in the conceptualization of person-centered care. We, however, do not find very significant differences. One potential reason is that the foundational work that led to the development of the final set of items included expert and cognitive interviews from India. Twenty-six items are common across the India and Kenya PCMC scales and could facilitate comparison across the two countries. While the validation of the PCMC tool with data from India demonstrates a similar set of items to that from the Kenya validation, there were a few notable exceptions. First, the item on “provider support anxieties” has been dropped from the current version of the scale because the question was identified as ambiguous based on feedback from in-country partners and reviewers of the original scale. Thus, this difference is not based on the empirical analysis, as it could have been retained based on its loading.

Second, the three items related to health facility environment—water, electricity, and crowding—that were included in the Kenya “Supportive care” sub-scale do not hang well with the rest of the items in India validation; therefore, they have been removed from this version of the scale. It is unclear why these items load much poorer in India than in Kenya. One reason is the difference in distribution in this sample, which affects the factor loading. Another potential reason is that this sample is from high-volume delivery facilities, and perceptions of person-centered care may be more closely related to the interpersonal relationships than to the broader health facility environment in this context. It should be noted that in the Kenya validation, “crowding” loaded well in urban samples, but not in rural samples. The majority of women in this sample were from rural areas of Uttar Pradesh. Therefore, the importance of health facility environment factors in influencing person-centered care may be more related to urban/rural context as opposed to Kenya vs. India. Also, the rewording of the question on availability of “water” to “drinking water” in India may have affected its relationship to the other items in the scale.

Since publishing the 30-item scale in Kenya, we have had discussions with various experts on whether to include the health facility environment items as part of the scale. While we have felt that the health facility environment is an important aspect of person-centered care, supported by a recent qualitative evidence synthesis on respectful maternity care [10], others have argued the health facility environment influences PCMC but is not necessarily part of it. The poor loading of these health facility environment related items might support this counter argument. Regardless, it is interesting to note the nuances of what is important to person-centered care across different contexts and cultures.

Third, the item on “being asked for bribes,” which was dropped from the Kenya scale has been retained in the India scale. The role of bribes is central to the mistreatment literature in India, as past studies have identified that poor and disadvantaged women, in particular, are asked to pay bribes in order to receive care [23, 39]. In cognitive interviews for the present study, women and providers in Kenya were both much less likely to report facility staff asking for bribes. However, in line with extant literature, bribes were much more common in cognitive interviews in India. It is therefore not surprising that this indicator would be retained in the India PCMC scale. The question used in the scale to get at bribes is “Did the doctors, nurses or other staff at the facility ask you or your family for money other than the official cost?”. But the complexity of the issue of bribes in this context is highlighted in other qualitative work by our team. These findings show women sometimes give providers tips, without being asked, when they feel providers are delaying care for them because they want bribes, even when this might not be the case; or when they are happy with the outcome of their pregnancies. In such cases they may be unhappy if the provider refuses to take the money. This suggests more than one question will be needed to unpack what is considered a bribe and what is not.

The differences across India and Kenya highlight the need for careful consideration of which items are most relevant across contexts as well as attention to the wording of items for different contexts. Validation in other settings is needed, and application of the full set of items in other parts of India followed by psychometric analysis will help to develop a tool that can be applied to all of India. But, given that not every study has the capacity to go through the process of validating a tool to use in the study, we believe the PCMC scale can be reasonably used across settings. It appears the 30-item version works well in settings like Kenya and the 27-item version works well in settings like Uttar Pradesh, India. Researchers and practitioners could therefore choose the version of the scale they believe will work best in their setting. However, given none of the samples used in the validation was nationally representative, it might be best to administer the full set of items if the length is not a concern. Analysis could then be conducted to assess whether the full set or only a subset will work best as a scale.

As in any study, there are a number of limitations. First, it should be noted that the sample is not generalizable to all of India—or even all of Uttar Pradesh. Women were recruited from public facilities that are all high volume (> 200 deliveries/month). Although we recruited women from a variety of facility levels, including primary health care centers and community health care centers, in addition to district hospitals, the sample excludes women who attend private and lower volume facilities and those who do not make it to a facility. The sample is however representative of women in Uttar Pradesh who gave birth in public health facilities, which represents 44% of all births and 66% facility-based births in Uttar Pradesh [16]. According to the recent National Family Health Survey (NFHS) survey (2015–16), among women who gave birth in the last 5 years in public facilities, about 86% resided in rural areas; 16% were Muslim and 84% were Hindus. Also, 99% were married, with an average age of 25 years and an average parity of 2 children; 56% had less than a secondary education and almost all (99.7%) belonged to the most vulnerable castes (Other backward caste (OBC) and Scheduled caste/Scheduled tribe (SCST)) [16]. This is similar to the characteristics of our sample which included 85% rural residents, 17% Muslim and 83% Hindi, with 99% married, average age of 25 years and parity of 2; and 47% with less than secondary education and majority (83.5%) from the most vulnerable castes. The characteristics of our sample are also somewhat similar to the general population of Uttar Pradesh, which is 73% rural, 18% Muslim and 81% Hindu, and 78% belong to the most vulnerable castes [16]. These similarities suggest that the PCMC scale may be applied to Uttar Pradesh, but it is possible that the factor structure may differ across different sub-populations within India, as was found in Kenya [8]. Nonetheless, given that data from two settings in Kenya and India all show one dominant factor, we believe the single multidimensional scale is likely stable across settings.

In addition, the data are self-reported, thus subject to recall bias and social desirability bias. Women may not clearly remember their experiences during their labor, and their recall may also be clouded by the outcome of their deliveries (although women who had an infant death were excluded). Recall is however likely not be a major issue with this sample given interviews occurred within 48 h of delivery. Social desirability bias on the other hand is a bigger issue as the interviews occurred in the post-natal ward. Prior research suggests women are more likely to report more positively on their experiences when interviewed close to the time of delivery and within the health facility [8, 28, 29]. Studies in India have also shown that women tend to report more positively in interviews when compared to direct observations due to normalization of certain mistreatment [25]. Thus, the levels of PCMC found in this study likely overestimates the quality of actual levels of PCMC in the study facilities. Finally, the 27-item scale may be considered too long for facilities wishing to include the tool as part of quality improvement initiatives. We used a relaxed cut-off to retain items in this analysis because the goal was not item reduction, but to assess construct validity of comprehensive scale with high content validity. This conservative approach is acceptable in early stages of scale development [30]. Future studies may, however, wish to use a more data-driven approach in order to focus the scale on a smaller set of indicators.

Despite these limitations, this study contributes in a number of ways to existing literature on person-centered care and quality of care for maternity services. In particular, this study validated an existing PCMC scale in a new context and found significant overlap in indicators across India and Kenya, two very different contexts. The overlap should not be very surprising in the light of a recent qualitative evidence synthesis on respectful maternity care, which concluded that globally, women’s perspectives of what constitutes respectful maternity care are quite consistent [10]. The overlap in items suggests that this scale can be used across many different contexts to compare women’s experiences of care.

It is important to note that the items in this scale capture all but one of the 12 domains of respectful maternity care from the recent review [10]. That the PCMC tool captures all of these domains, except continuity of care, is not surprising given the initial work towards its development involved a review of the literature including that on mistreatment/disrespect and abuse/respectful maternity care. Thus, the person-centered maternity care scale is an effective tool for holistically measuring respectful maternity care and might be among the best tools currently available for this purpose. An additional question on continuity of care may however be needed to complete it. Future studies looking at person-centered care measures across the continuum of reproductive care, including family planning will also help improve continuity of care.

Finally, a recent review of quantitative studies on disrespect and abuse highlighted how differences in study tools as well as other methodological differences affect comparison of different studies, hence the need for more consistent methodologies if we are to be able to compare studies across settings (while taking into account key contextual differences) [39]. We believe the PCMC tool having so far been validated in three settings in two countries addresses one of the key steps towards responding to this call.

## Conclusions

This study presents the results of validating the PCMC scale in Uttar Pradesh, India. The PCMC scale provides a valuable tool for the growing number of quality improvement initiatives in India, and beyond. The scale may also be used to support policy and programmatic efforts to improve the quality of maternity care. Five years ago, the Government of India developed broad quality assurance guidelines for quality of care, and several years later issued a maternal and neonatal health care manual to guide providers about importance of patient-centered care during labor and delivery [40, 41]. However, it is unclear how to measure and evaluate women’s experiences of care across different facilities in India. This scale will help to bridge this gap. In addition, this scale will be valuable for assessing implementation of the WHO recommendations on intrapartum care for a positive childbirth experience [6]. Providing clear guidelines and standard measurement tools will help improve accountability of facilities, support providers/staff in understanding how to provide person-centered care, and ensure women’s voices, preferences, and values are front and center in the care they receive.

## Appendix

**Table 7:** Distribution of PCMC variables

PCMC variables	No.	%
How did you feel about the amount of time you waited? Would you say it was		
0 Very short	1347	66.7
1 Somewhat short	410	20.3
2 Somewhat long	177	8.8
3 Very long	84	4.2
During your time in the health facility did the doctors, nurses, or other health care providers introduce themselves to you when they first came to see you?		
0 No, none of them	1980	98.1
1 Yes, a few of them	35	1.7
2 Yes, most of them	2	0.1
3 Yes, all of them	1	0.0
Did the doctors, nurses, or other health care providers call you by your name?		
0 No, never	567	28.1
1 Yes, a few times	436	21.6
2 Yes, most of the time	371	18.4
3 Yes, all the time	644	31.9
Did the doctors, nurses, or other staff at the facility treat you with respect?		
0 No, never	143	7.1
1 Yes, a few times	299	14.8
2 Yes, most of the time	531	26.3
3 Yes, all the time	1045	51.8
Did the doctors, nurses, and other staff at the facility treat you in a friendly manner?		
0 No, never	92	4.6
1 Yes, a few times	358	17.7
2 Yes, most of the time	545	27.0
3 Yes, all the time	1023	50.7
Did you feel the doctors, nurses, or other health providers shouted at you, scolded, insulted, threatened, or talked to you rudely?		
0 No, never	1661	82.3
1 Yes, once	212	10.5
2 Yes, a few times	131	6.5
3 Yes, many times	14	0.7
Did you feel like you were treated roughly like pushed, beaten, slapped, pinched, physically restrained, or gagged?		
0 No, never	1967	97.5
1 Yes, once	31	1.5
2 Yes, a few times	17	0.8
3 Yes, many times	3	0.1
During examinations in the labor room, were you covered up with a cloth or blanket or screened with a curtain so that you did not feel exposed?		
0 No, never	526	26.1
1 Yes, a few times	115	5.7
2 Yes, most of the time	228	11.3
3 Yes, all the time	1149	56.9
4 Not applicable		
Do you feel like your health information was or will be kept confidential at this facility?		
0 No, never	324	16.1
1 Yes, a few times	444	22.0
2 Yes, most of the time	387	19.2
3 Yes, all the time	863	42.8
Did you feel like the doctors, nurses or other staff at the facility involved you in decisions about your care?		
0 No, never	1131	56.0
1 Yes, a few times	311	15.4
2 Yes, most of the time	255	12.6
3 Yes, all the time	321	15.9
4 Did not have to make any decisions		
During the delivery, do you feel like you were able to be in the position of your choice?		
0 No, never	360	17.8
1 Yes, for a short time	655	32.5
2 Yes, most of the time	418	20.7
3 Yes, all the time	585	29.0
Did the doctors, nurses or other staff at the facility speak to you in a language you could understand?		
0 No, never	16	0.8
1 Yes, a few times	131	6.5
2 Yes, most of the time	315	15.6
3 Yes, all the time	1556	77.1
Did the doctors, nurses or other staff at the facility ask your permission/consent before doing procedures on you?		
0 No, never	1475	73.1
1 Yes, a few times	282	14.0
2 Yes, most of the time	172	8.5
3 Yes, all the time	89	4.4
Did the doctors and nurses explain to you why they were doing examinations or procedures on you?		
0 No, never	1393	69.0
1 Yes, a few times	344	17.0
2 Yes, most of the time	174	8.6
3 Yes, all the time	107	5.3
Did the doctors and nurses explain to you why they were giving you any medicine?		
0 No, never	1162	57.6
1 Yes, a few times	400	19.8
2 Yes, most of the time	242	12.0
3 Yes, all the time	205	10.2
4 Did not get any medicine	9	0.4
Did you feel you could ask the doctors, nurses or other staff at the facility any questions you had?		
0 No, never	265	13.1
1 Yes, a few times	437	21.7
2 Yes, most of the time	543	26.9
3 Yes, all the time	773	38.3
Did the doctors and nurses at the facility talk to you about how you were feeling?		
0 No, never	817	40.5
1 Yes, a few times	776	38.5
2 Yes, most of the time	326	16.2
3 Yes, all the time	99	4.9
Did the doctors, nurses or other staff at the facility try to understand your anxieties?		
0 No, never	456	22.6
1 Yes, a few times	667	33.1
2 Yes, most of the time	442	21.9
3 Yes, all the time	453	22.4
4 I did not have any anxieties or fears		
When you needed help, did you feel the doctors, nurses or other staff at the facility paid attention?		
0 No, never	80	4.0
1 Yes, a few times	403	20.0
2 Yes, most of the time	634	31.4
3 Yes, all the time	901	44.6
Do you feel the doctors or nurses did everything they could to help control your pain?		
0 No, never	182	9.0
1 Yes, a few times	478	23.7
2 Yes, most of the time	759	37.6
3 Yes, all the time	599	29.7
Were you allowed to have someone you wanted (outside of staff at the facility, such as family or friends) to stay with you during labor?		
0 No, never	157	7.8
1 Yes, a few times	92	4.6
2 Yes, most of the time	295	14.6
3 Yes, all the time	1474	73.0
4 I did not want someone to stay with me		
Were you allowed to have someone you wanted to stay with you during delivery?		
0 No, never	175	8.7
1 Yes, a few times	79	3.9
2 Yes, most of the time	270	13.4
3 Yes, all the time	1494	74.0
4 I did not want someone to stay with me		
Do you think there was enough health staff in the facility to care for you?		
0 No, never	36	1.8
1 Yes, a few times	320	15.9
2 Yes, most of the time	696	34.5
3 Yes, all the time	966	47.9
Did you feel the doctors, nurses or other staff at the facility took the best care of you?		
0 No, never	69	3.4
1 Yes, a few times	444	22.0
2 Yes, most of the time	831	41.2
3 Yes, all the time	674	33.4
Did you feel you could completely trust the doctors, nurses or other staff at the facility with regards to your care?		
0 No, never	55	2.7
1 Yes, a few times	144	7.1
2 Yes, most of the time	453	22.4
3 Yes, all the time	1366	67.7
Thinking about the labor and postnatal wards, did you feel the health facility was croweded?		
0 No, never	427	21.2
1 Yes, a few times	803	39.8
2 Yes, most of the time	563	27.9
3 Yes, all the time	225	11.1
Thinking about the wards, washrooms and the general environment of the health facility, will you say the facility was very clean, clean, dirty, or very dirty?		
0 Very dirty	355	17.6
1 Dirty	386	19.1
2 Clean	118	5.8
3 Very clean	1159	57.4
Was there water in the facility?		
0 No, never	264	13.1
1 Yes, a few times	48	2.4
2 Yes, most of the time	222	11.0
3 Yes, all the time	1484	73.5
Was there electricity in the facility?		
0 No, never	8	0.4
1 Yes, a few times	135	6.7
2 Yes, most of the time	920	45.6
3 Yes, all the time	955	47.3
In general, did you feel safe in the health facility?		
0 No, never	30	1.5
1 Yes, a few times	45	2.2
2 Yes, most of the time	226	11.2
3 Yes, all the time	1717	85.1
Did the doctors, nurses or other staff at the facility ask you or your family for money other than the official cost?		
0 No, never	1328	65.8
1 Yes, a few times	526	26.1
2 Yes, most of the time	156	7.7
3 Yes, all the time	8	0.4
Were you or your family asked to buy anything from outside the health facility for your care?		
0 No, never	1499.00	74.3
1 Yes, a few times	441	21.9
2 Yes, most of the time	69	3.4
3 Yes, all the time	9	0.4
Total N	2018	100.0

## Abbreviations

CA: Communication and Autonomy
DR: Dignity and Respect
HFE: Health Facility Environment
KMO: Kaiser-Meyer-Olkin
PCMC: Person-centered maternity care
SC: Supportive Care
WHO: World Health Organization
